# Purinergic Signaling to Terminate TLR Responses in Macrophages

**DOI:** 10.3389/fimmu.2016.00074

**Published:** 2016-03-02

**Authors:** Kajal Hamidzadeh, David M. Mosser

**Affiliations:** ^1^Department of Cell Biology and Molecular Genetics, The Maryland Pathogen Research Institute, University of Maryland, College Park, MD, USA

**Keywords:** adenosine, autoimmunity, ATP, CD39, CD73, glycolysis, IFN-γ

## Abstract

Macrophages undergo profound physiological alterations when they encounter pathogen-associated molecular patterns (PAMPs). These alterations can result in the elaboration of cytokines and mediators that promote immune responses and contribute to the clearance of pathogens. These innate immune responses by myeloid cells are transient. The termination of these secretory responses is not due to the dilution of stimuli, but rather to the active downregulation of innate responses induced by the very PAMPs that initiated them. Here, we describe a purinergic autoregulatory program whereby TLR-stimulated macrophages control their activation state. In this program, TLR-stimulated macrophages undergo metabolic alterations that result in the production of ATP and its release through membrane pannexin channels. This purine nucleotide is rapidly hydrolyzed to adenosine by ectoenzymes on the macrophage surface, CD39 and CD73. Adenosine then signals through the P1 class of seven transmembrane receptors to induce a regulatory state that is characterized by the downregulation of inflammatory cytokines and the production of anti-inflammatory cytokines and growth factors. This purinergic autoregulatory system mitigates the collateral damage that would be caused by the prolonged activation of macrophages and rather allows the macrophage to maintain homeostasis. The transient activation of macrophages can be prolonged by treating macrophages with IFN-γ. IFN-γ-treated macrophages become less sensitive to the regulatory effects of adenosine, allowing them to sustain macrophage activation for the duration of an adaptive immune response.

## Introduction

The central role that macrophages play in host defense has been well described and thoroughly studied. These remarkable cells can change their physiology in response to diverse environmental stimuli and become potent antimicrobial effectors. This property has been loosely called an “activation” response, and the receptors that induce this response are generally called pattern recognition receptors (PRRs) (1). More recently, the role of macrophages in mitigating inflammatory responses and contributing to the resolution of inflammation has become an area of intense study (2, 3). It is clear that the very cell type that could be a potent inducer of inflammatory pathology could be equally effective at reversing this pathology. The remarkable plasticity of macrophages allows this cell to be a primary mediator of homeostasis in the host (4, 5).

Given the remarkable differences in the physiologies of the various macrophage subsets, efforts are underway to characterize each. These characterizations would theoretically allow the identification of each macrophage subtype in tissue during immunity or immunopathology. However, studies to identify definitive biochemical differences between inflammatory M1 macrophages and anti-inflammatory regulatory macrophages (R-Mϕ) have been surprisingly underdeveloped. The *in vitro* transcriptional responses of the so-called M1 macrophages following their exposure to a variety of TLR ligands, such as LPS or to bacteria themselves, have been reported (6–11). These studies have begun to reveal the molecules that macrophages express and the products they secrete in response to inflammatory stimuli. However, most of these studies lack a careful kinetic analysis of transcriptional responses over time. Therefore, we are left with “snap-shots” of transcriptional responses to stimuli, rather than a motion picture of the sequential transcriptional program these stimuli induce. The transcriptional responses of anti-inflammatory macrophages have also been described (12, 13), but again these studies generally selected only a single time to analyze macrophage transcripts. In this review, we propose that one of the difficulties in identifying definitive biochemical differences between the various macrophage cell populations is due to the transient nature of the inflammatory response of macrophages to stimuli and the compensatory regulatory changes that accompany this activation. We describe an intrinsic program where the metabolic alterations that allow for the production of inflammatory cytokines and mediators are the very alterations that give rise to the anti-inflammatory macrophage phenotype. This autoregulatory response depends on the generation of endogenous ATP by macrophages, which initiates a purinergic signaling cascade to terminate the inflammatory response to innate stimuli, resulting in a transient state of activation. Therefore, the time when one measures the transcriptional responses of macrophages to TLR stimuli is critical. We also propose that this transient state of macrophage stimulation can be prolonged and accentuated in individuals undergoing cell-mediated immune responses. This is due to a novel activity of IFN-γ, which interferes with the stimulus-dependent upregulation of adenosine receptors to block purinergic autoregulatory responses. In this way, IFN-γ prevents the transition to a regulatory macrophage and prolongs the activation response.

## Metabolic Alterations Induced by the Ligation of Macrophage Pattern Recognition Receptors

When macrophages encounter pathogen-associated molecular patterns (PAMPs) or damage-associated molecular patterns (DAMPs) they undergo dramatic changes in their metabolism and increase their rate of aerobic glycolysis. An increase in glucose uptake by these cells results in an accumulation of lactate in M1 macrophages (12). In contrast to M1 macrophages stimulated by PAMPs, alternatively activated macrophages exposed to IL-4 or IL-13 undergo oxidative phosphorylation and electron transport. The metabolic alterations associated with M1 macrophage polarization are believed to provide short-term, immediate access to energy for innate immune functions, whereas alternative activation is thought to provide a more stable long-term metabolism to support prolonged processes associated with wound healing (14). Recent work suggests that these metabolic alterations not only accompany differential activation but also promote the polarized responses of M1 and M2 macrophages (15). The rapid alterations in metabolism that M1 macrophages undergo are thought to allow these cells to produce the cytokines and mediators associated with host defense [reviewed in Ref. (14)]. However, the increase in glycolysis by M1 macrophages results in an increase in the production of intracellular ATP by stimulated macrophages. A portion of cytosolic ATP generated by M1 macrophages is released into the extracellular milieu *via* pannexin-1 channels. The addition of inhibitors of either glycolysis or pannexin channels prevents ATP release from macrophages (16). This released ATP is rapidly captured and catabolized to adenosine by M1 macrophages, allowing them to transition from an inflammatory to a regulatory phenotype. Thus, the very metabolic alterations that allow M1 macrophages to promote immune responses can also prevent these cells from causing immunopathology.

## The Macrophage Ectoenzymes, CD39 and CD73

Purinergic signaling molecules released as a result of metabolic alterations, cell death, or tissue damage can have profound effects on macrophage activation. ATP concentrations in human plasma are typically in the nanomolar range (17) but can rise to the micromolar range under inflammatory conditions (18). ATP is constitutively released from resting parenchymal cells, and the levels are intrinsic to the tissue in which the cells reside (19). ATP release from resting macrophages is quite low, but this release is substantially increased upon TLR stimulation (16). The ATP that is released by macrophages is catabolized by macrophages in a coordinated two-step process. First, ATP is hydrolyzed to AMP by the macrophage surface ectoenzyme CD39 (E-NTPDase1) in a Ca^2+^- and Mg^2+^-dependent manner (20). AMP is then rapidly converted to adenosine by the surface Ecto5′NTase, CD73 (21). The expression of these two enzymes by macrophages can therefore determine the concentration of adenosine in the extracellular milieu immediately surrounding the macrophage.

CD39 and CD73 expression on macrophages can change depending on the macrophage activation state. In hypoxic conditions, CD39 and CD73 function is enhanced approximately sixfold (22), whereas prolonged cultivation of macrophages in complete medium appears to downregulate CD73 expression. CD39 is more highly expressed than CD73 on bone marrow derived macrophages, and this expression pattern remains relatively constant after a brief exposure of these cells to LPS. M1 macrophages have been reported to exhibit a modest decrease in the expression of both CD39 and CD73 (23), while M2 macrophages express higher levels of both (23). These results suggest that macrophages may regulate the catabolism of ATP in order to modulate their inflammatory profile. It has also been shown that CD39 is transcriptionally regulated by the cAMP/CREB second messenger pathway that can be induced following GPCR ligation (24, 25). This suggests a positive feedback loop where adenosine signaling upregulates CD39 to generate more adenosine. Overall, this work suggests that the increased expression of either/both of these cell surface enzymes can result in an amplification of the purinergic signaling pathway in macrophages.

We recently demonstrated that the addition of exogenous adenosine or ATP to macrophages can induce these cells to assume an anti-inflammatory phenotype (16) characterized by a decreased production of inflammatory cytokines and an increased expression of angiogenic factors and anti-inflammatory cytokines (12). We further demonstrated that the hydrolysis of self-released (endogenous) ATP *via* macrophage CD39 allows that cell to transition from an inflammatory to an immunoregulatory state (16). Macrophages derived from CD39 knockout bone marrow fail to catabolize ATP following LPS stimulation. As a result, the production of inflammatory cytokines is sustained for up to 24 h poststimulation, whereas wild-type macrophages stop synthesizing these cytokines after a few hours (16). Similarly, the pharmacological inhibition of CD39 activity, using the chemical inhibitor POM-1, made macrophages hyperinflammatory with increased TNF and IL-12p40 production over the course of at least 16 h (16). It appears that of the two ectoenzymes involved in ATP hydrolysis, CD39 has more profound effects than CD73, presumably because the conversion of AMP to adenosine can occur in the absence of CD73. It was recently demonstrated that an inhibitor of CD73 did not have a substantial role in macrophage polarization (26).

The ability of macrophages to transition to an immunoregulatory state is key in controlling pathology in an LPS model of endotoxemia. Our lab results and others have shown that CD39 on myeloid cells can decrease mortality in mouse models of sepsis (16, 27), and that the addition of CD39 knockout macrophages can increase mortality in this model (16). CD73 has also been demonstrated to be protective in mouse models of sepsis (28).

## The Receptors for Adenosine

Macrophages respond to adenosine *via* four transmembrane G-protein-coupled receptors: A1R, A2aR, adenosine 2b receptor (A2bR), and A3R (29). The A1 and A3 receptors are coupled to the G_i_ family of proteins resulting in decreased cAMP upon stimulation. A2a receptors are high affinity Gα_s_-coupled receptors that increase intracellular cAMP (30, 31). Similarly, the low-affinity A2b receptors can signal through Gα_s_ or G_q_ proteins, also resulting in increased cAMP (30, 32). When coupled to TLR stimulation, adenosine promotes the transition from an inflammatory to a regulatory macrophage (4). Adenosine is known to be immunosuppressive in macrophages as adenosine treatment leads to increased IL-10 production and decreased TNF and IL-12 production (16). We recently performed high-throughput RNA sequencing on macrophages stimulated with LPS in the presence or absence of adenosine. Macrophages stimulated with LPS in the presence of adenosine upregulated 501 transcripts relative to LPS alone and downregulated 610 transcripts. Many of the genes that were upregulated were involved in cell growth and neovascularization, whereas genes involved in inflammation were most potently downregulated by the presence of adenosine (12, 13). Adenosine signaling through its Gα_s_-coupled receptors also leads to increased IL-10 production *via* posttranscriptional mechanisms (33). Adenosine is thought to inhibit the production of the inflammatory cytokine TNF by signaling through both the A2a and A2b receptors (34).

Although signaling through these GPCRs modulates levels of cAMP within cells, the role of the cAMP/PKA pathway in the regulation of inflammatory cytokines by adenosine receptor signaling remains somewhat unclear. One group has indicated that the decrease in macrophage TNF production after exposure to adenosine is due to a cAMP/PKA-independent pathway, which likely involves phosphatases (35). However, others have shown that cAMP/PKA levels are inversely correlated with TNF production (36). Thus, it is possible that while cAMP itself, mainly investigated in the form of 8-bromo-cAMP, can downregulate TNF production in macrophages, adenosine may also work by additional mechanisms that have not yet been fully defined. It was shown that the A2bR interacts with NF-κB in order to inhibit it, and that A2bR knockout macrophages secrete less IL-10 and more IL-12 and TNF (37).

The adenosine receptors have been implicated in the pathology of a variety of diseases. These receptors are widely expressed in the brain, heart, spleen, muscle, and lung (38, 39). In fact, their widespread expression is one of the challenges of developing therapeutics targeting the receptors with specificity. Studies have implicated a role for both A2aR and A2bR in diabetes as they are involved in gluconeogenesis and glucose homeostasis as a result of increased cAMP (40–42). There is also therapeutic anti-inflammatory potential for A2aR agonists in ischemia reperfusion injury (43). In atherosclerosis, A2aR and A2bR both play a role in reducing foam cell formation, which is a feature of this disease (44, 45). However, it has been shown that the lack of A2aR has a protective effect in a mouse model of hypercholesterolemia because macrophages remain inflammatory and are able to reduce atherosclerotic lesions (46). Adenosine receptors also play a role in wound healing and contribute to cytokine production by macrophages of patients with chronic obstructive pulmonary disease (29, 47).

## IFN-γ and the Prolongation of the Macrophage Activation Response

Priming macrophages with IFN-γ prior to TLR stimulation results in profound changes in their physiology and dramatically accentuates their inflammatory responses (48, 49). Macrophages exposed to IFN-γ not only make greater amounts of inflammatory cytokines but also produce them for prolonged periods of time (50). In this way, IFN-γ prolongs the activation response to promote host defense against intracellular pathogens (51). The activation of macrophages, however, comes at a cost. Inflammatory macrophages exhibiting an “IFN signature” are observed in rheumatoid arthritis, multiple sclerosis, and many other autoimmune diseases, indicating that IFN-γ contributes to autoimmune pathogenesis by promoting chronic macrophage activation (52, 53). Although the ability of IFN-γ to enhance the inflammatory potential of TLR-activated macrophages is a well-known phenomenon, the mechanism(s) whereby IFN-γ affects the intrinsic regulation of macrophage activation remain to be determined. We recently identified a novel mechanism whereby IFN-γ sustains macrophage inflammatory responses, by attenuating their sensitivity to extracellular adenosine (50).

Following TLR stimulation, macrophages dramatically upregulate their expression of receptors for adenosine. The A2bR, in particular, is upregulated more than 20-fold in response to virtually any of the TLR ligands (50). The molecular mechanism(s) of A2bR upregulation remain to be determined, but the upregulation of adenosine receptors in response to TLR stimulation enhances macrophage sensitivity to adenosine and leads to the induction of the immunoregulatory phenotype. IFN-γ priming of macrophages signals through STAT1 to prevent adenosine receptor induction. This decreases macrophage sensitivity to adenosine and delays the transition of macrophages to a regulatory phenotype. This prolongs the production of inflammatory cytokines such as TNFα and IL-12. Thus, we propose a novel mechanism whereby IFN-γ contributes to host defense, by desensitizing macrophages to the immunoregulatory effects of adenosine. This mechanism overcomes the transient nature of TLR activation and prolongs the antimicrobial state of the classically activated macrophage.

## Summary

**Figure 1:**
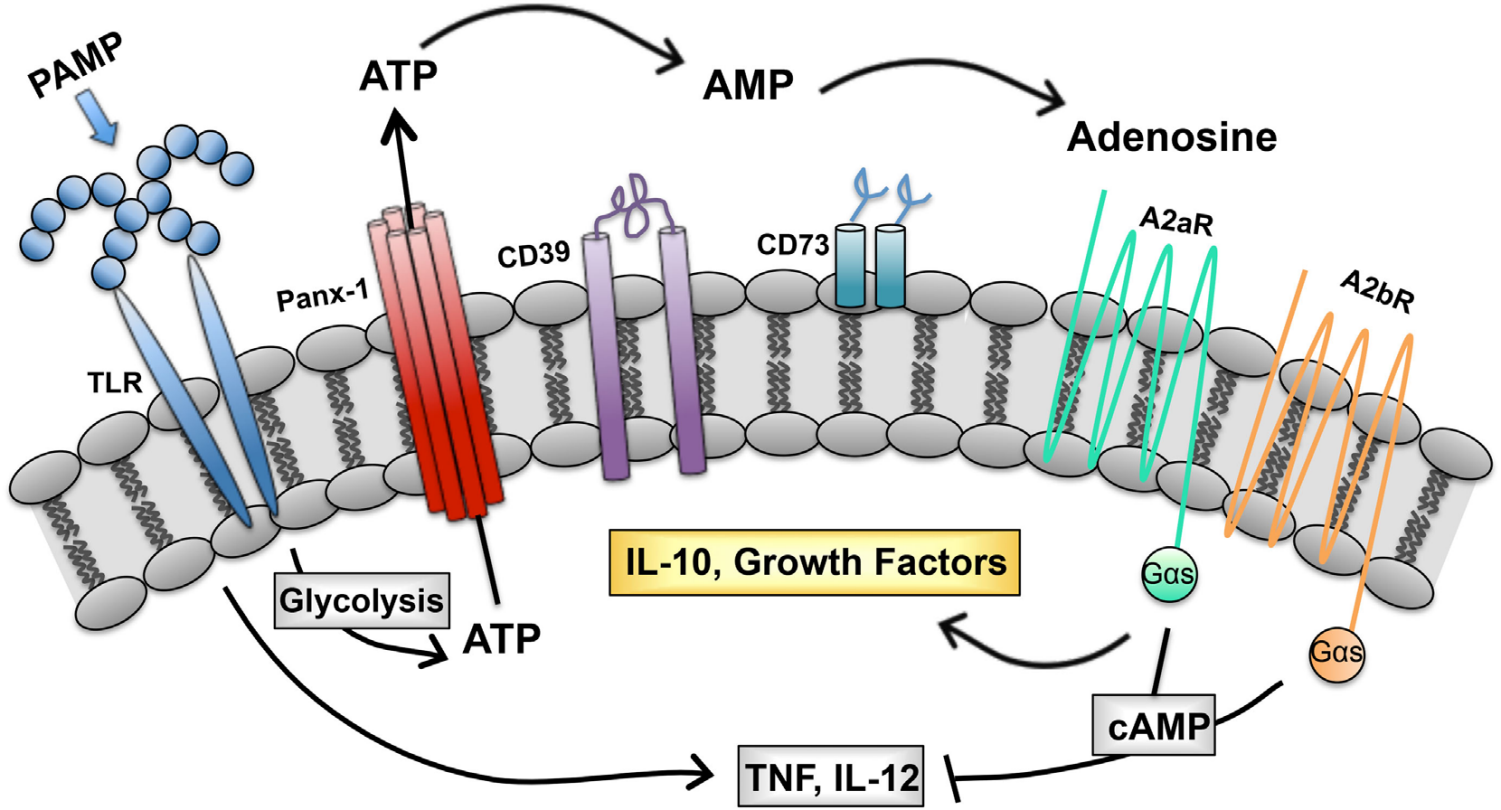
**Purinergic autoregulatory signaling in macrophages**. Intracellular ATP, generated in response to pathogen-associated molecular patterns (PAMPS), is released from macrophages and converted to adenosine by the concerted action of CD39 and CD73. Adenosine signals through seven transmembrane receptors to terminate the production of inflammatory cytokines and to promote the production of IL-10 and growth factors (54).

We describe a purinergic-based autoregulatory program that terminates inflammatory responses of TLR-stimulated (M1) macrophages. When macrophages are so stimulated, they undergo metabolic alterations that result in ATP generation and release through pannexin channels. Extracellular ATP is rapidly hydrolyzed to adenosine by CD39 and CD73, two ectoenzymes on the macrophage surface. Adenosine generated in this way binds to macrophage adenosine receptors to initiate a signaling pathway that terminates the synthesis of many inflammatory cytokines and induces the synthesis of regulatory transcripts (Figure 1). In this way, the overexuberant activation of macrophages is avoided. We propose that this program is in place to prevent the pathological consequences associated with chronic macrophage activation. We suggest that there are many ways to exploit this program to manipulate the phenotype of macrophages. The overexpression of CD39 and CD73 would be predicted to accelerate adenosine production by macrophages and promote a rapid regulatory transition. Drugs to prevent ectoenzyme downregulation may represent a new class of anti-inflammatory therapeutics. Similarly, drugs to induce adenosine receptor upregulation or prevent their downregulation may be developed as a way to interrupt macrophage-mediated inflammation. Conversely, targeting macrophage CD39 would be predicted to prevent this regulatory transition and promote the more efficient killing of intracellular pathogens by macrophages.

## Author Contributions

The ideas expressed in this review were developed by both authors who contributed equally to the writing of this review.

## Conflict of Interest Statement

DM declares partial ownership in LeukoSight, Inc., a company developing a line of anti-inflammatory therapeutics. KH has no conflict of interest to declare.
